# Study on the Material Basis of Houpo Wenzhong Decoction by HPLC Fingerprint, UHPLC-ESI-LTQ-Orbitrap-MS, and Network Pharmacology

**DOI:** 10.3390/molecules24142561

**Published:** 2019-07-14

**Authors:** Juyuan Luo, Gongsen Chen, Donghan Liu, Yan Wang, Qi Qi, Haiyan Hu, Pengyue Li, Jie Bai, Shouying Du, Yang Lu, Yuming Wang, Cun Liu

**Affiliations:** 1 School of Chinese Materia Medica, Beijing University of Chinese Medicine, Yangguang South Avenue, Fangshan District, Beijing 102488, China; 2 Yifan Pharmaceutical Co., Ltd., Lin’an 311300, China

**Keywords:** Houpo Wenzhong decoction, UHPLC-ESI-LTQ-Orbitrap-MS, network pharmacology, material basis

## Abstract

Houpo Wenzhong Decoction (HWD) as a classical prescription has been widely used for hundreds of years. However, the quality of HWDs is difficult to control because of its herb materials from different regions. It is a new idea to use HPLC fingerprints, LTQ-ESI-Orbitrap-MS, and network pharmacology in combination to screen common components (CCs) as potential quality control indicators. In this paper, the fingerprints of HWDs were established, which were used to determine HWDs compounded from different sources of traditional Chinese medicines (TCMs). Through the similarity analysis, 45 CCs were selected. UHPLC-LTQ-ESI-Orbitrap-MS was used to carry out the chemical composition analysis of HWD. Seventy-three chemical constituents were distinguished, and 30 CCs were identified. Through network pharmacology, networks of candidate CCs, diseases, and candidate targets were constructed. Finally, four CCs were screened as potential active ingredient markers of HWD, and a method for content determination of these four components was established. A rapid, reasonable, and effective method for quality evaluation and control of HWDs was established. It provides a reference for the further development and research of HWDs and a new way of thinking for the research of other Chinese medicine prescriptions.

## 1. Introduction

Houpo Wenzhong decoction (HWD), as a classical Chinese medicine prescription, has been widely used in clinical practice for nearly 800 years, since the Jin Dynasty, and has been used extensively for the treatment of gastric pain (GP) of the spleen-stomach weak and cold type [1,2,3], functional dyspepsia (FD) [4,5,6,7], chronic gastritis (CG) of the spleen-stomach weak and cold type [8,9,10,11], mesenteric lymphadenitis [12], diarrhea, [13] and functional recurrent abdominal pain [14]. It is composed of *Magnolia officinalis Cortex*, *Citri Reticulatae Pericarpium*, *Radix aucklandiae*, *Glycyrrhiza uralensis Fisch*, *Semen Alpiniae Katsumadai*, *Poria cocos*, and *Rhizoma Zingiberis*. Since there are seven herbs in the prescription, quality control is difficult, especially when mixing and decocting traditional Chinese medicines (TCMs) from different origins.

Traditional Chinese medicine (TCM) prescriptions are usually made up of a variety of TCMs, and their quality are easily affected by factors such as origin [15], climate [16], growth years [17], harvesting time, and processing methods [18]. In order to ensure that they have stable clinical therapeutic effect, reasonable quality control is very necessary. HPLC fingerprint chromatography provides a more comprehensive approach for quality control of HWDs because of its wide applicability, high sensitivity, high efficiency, and easy operation [19,20]. Similarity analysis, as a significant evaluation index of the fingerprint of TCMs, can reflect the similarity of the response ratio of chemical components and find common components (CCs) among different batches of samples. Therefore, it is very reliable to find CCs by fingerprint and similarity analysis to represent the common characteristics of multi-origin and multi-batch prescription.

Although CCs can be obtained by fingerprint, the material basis of the HWDs is not clear, the structural information of CCs is not known, and whether they can be used to evaluate the quality of HWDs cannot be easily determined. Therefore, it is indispensable to identify the material basis of HWDs, especially the CCs. LTQ-ESI-Orbitrap-MS not only can provide multi-stage MS analysis information, accurate mass charge ratios (*m*/*z*), and structural information of fragment ions, but also can confirm the structure of trace components in complicated samples [21,22,23,24,25,26]. LTQ-ESI-Orbitrap-MS is very suitable for analyzing and identifying the material basis of HWDs because of its rapid, sensitive, and reliable detection [27]. This laid a solid foundation for further research on quality control of HWD.

Peng et al. [28] controlled the quality of Shengjiang Xiexin decoction by simultaneous determination of 14 active components by UFLC-MS/MS. However, it is not clear whether the indicators are related to drug efficacy. In addition, although some indicators are active ingredients, it is doubtful whether they correspond to the symptoms of the prescription. Network pharmacology uses the network model to express and study the interaction between drugs, components, diseases, and targets. It not only can reveal the complex mechanism of TCM in the treatment of diseases [29], but also can find active compounds [30,31]. Therefore, after the information of the CCs are identified by fingerprint and LTQ-ESI-Orbitrap-MS, potential active ingredients can be screened by network pharmacology as quality control indicators to evaluate the quality of HWD.

In summary, a new idea for the quality control of complex prescriptions is put forward in this paper. CCs were screened through HPLC fingerprints from multiple batches and multiple origins, and identified by LTQ-ESI-Orbitrap-MS, then the identified CCs were used as candidate active ingredients to carry out network pharmacological analysis together with the diseases treated by the prescription. Combined with network pharmacological analysis results, professional knowledge, and literature verification, the potential active ingredient markers of quality control were initially determined and a corresponding quantitative determination method was established. Finally, fingerprint and potential active ingredients markers were used as quality control means to evaluate the TCM prescriptions. This method combines three mature modern research methods to find trustworthy potential active ingredients to control the quality of prescriptions. Not only to give full play to the respective advantages of the three technologies, but also to establish a reliable, fast, and scientific quality control method, which may provide a new reference for the study of TCM prescription.

## 2. Results

### 2.1. Optimization of Extraction Conditions for Samples of Fingerprint Analysis and UHPLC-ESI-LTQ-Orbitrap-MS Analysis

Using the peak area of the common peaks as an index, ultrasonic time, amount of solvent, and dissolution solvent were investigated. The results show that there was no significant difference in different amounts of 70% aqueous methanol (2.5 mL, 5 mL, and 10 mL) and different ultrasonic time (15 min, 30 min, and 45 min) to dissolve lyophilized powder of HWDs. However, there were more liposoluble constituents in HWDs, such as magnolol, honokiol, alpinetin, and so on. With the increase of methanol concentration, the peak area of most components showed a significant upward trend in varying degrees, while the peak area of water-soluble components did not decrease. There was no diversity between 50% aqueous methanol and 70% aqueous methanol. Therefore, the optimum condition of lyophilized powder dissolution was as follows: adding 5 mL 70% aqueous methanol precisely and ultrasonic extraction for 15 min.

### 2.2. Optimization of Chromatographic Conditions for Fingerprint Analysis

The composition of the mobile phase, column temperature, and detection wavelength were investigated. Different organic phases (methanol and acetonitrile) and different aqueous phases (water and 0.1% phosphoric acid) were compared. When acetonitrile 0.1% phosphoric acid was used as the mobile phase, more chemical information, better separation effect, and higher responses were obtained. The wavelength was selected from 190 to 400 nm. There were many interferences of solvent peaks when the wavelength was less than 230 nm. However, when the wavelength was greater than 230 nm, the response of peak decreased gradually. Thus, 230 nm was chosen as the detection wavelength. The column temperatures of 25 °C, 30 °C, and 35 °C were investigated. The results showed that the separation of chromatographic peaks was better at 30 °C.

### 2.3. Validation of the Fingerprint Method

The hesperidin with a large peak area in the chromatogram was set as the reference peak (peak 22), and the relative retention times (RRTs) and relative peak areas (RPAs) were calculated. Verification of the fingerprint method by using RRTs, RPAs, and fingerprint similarity as indicators.

The precision refers to the repeated injection of the same sample six times, which is a prerequisite for ensuring measurement accuracy. The relative standard deviations (RSDs) of RRTs and RPAs of common peaks were less than 0.01% and 1.77%, respectively. The similarities of six chromatograms were all above 0.997.

The repeatability means that the same operator prepared six samples for testing in the same laboratory and in the same way. The RSDs of RRTs and RPAs of the common peaks were less than 0.03% and 1.57%, respectively. The similarities of six chromatograms were 0.999.

The stability of the same sample was evaluated at multiple time points, indicating the ability of the sample to remain unchanged over 24 h. The RSDs of RRTs and RPAs of the common peaks were less than 0.02% and 2.01%, respectively. The similarities of six chromatograms were 0.990.

The above results demonstrate that the sample was stable during the experiment and the fingerprint analysis method of HWDs is reliable and reproducible.

### 2.4. Similarity Analysis

**Figure 1:**
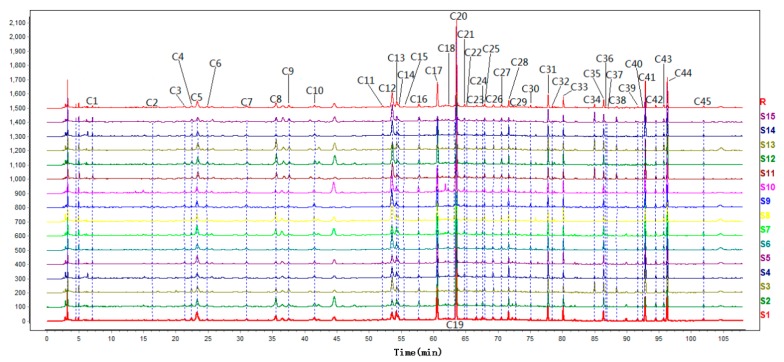
UHPLC chromatograms of 15 batches of Houpo Wenzhong Decoction (HWD) samples from S1–S15. The 45 common peaks (C1–C45) are marked.

Fifteen HWD samples were prepared by TCM from different origins and these 15 chromatograms were imported into SES (Figure 1). Common peaks with a peak area of more than 0.01% of the total peak area were obtained in the matching. Forty-five common peaks (Peaks C1–C45) were labeled and a reference spectrum was generated (R). SES was used to calculate the similarities between batches of sample chromatograms and the reference spectrum.

From the results, it is not difficult to see that the similarities between the fingerprints of the 15 batches of samples and the simulative median chromatograms were all greater than 0.920, which shows that the main chemical constituents of the prescription and most TCMs from the different areas had no significant influence overall.

### 2.5. Chemical Profiling by UHPLC-ESI-LTQ-Orbitrap-MS

**Figure 2:**
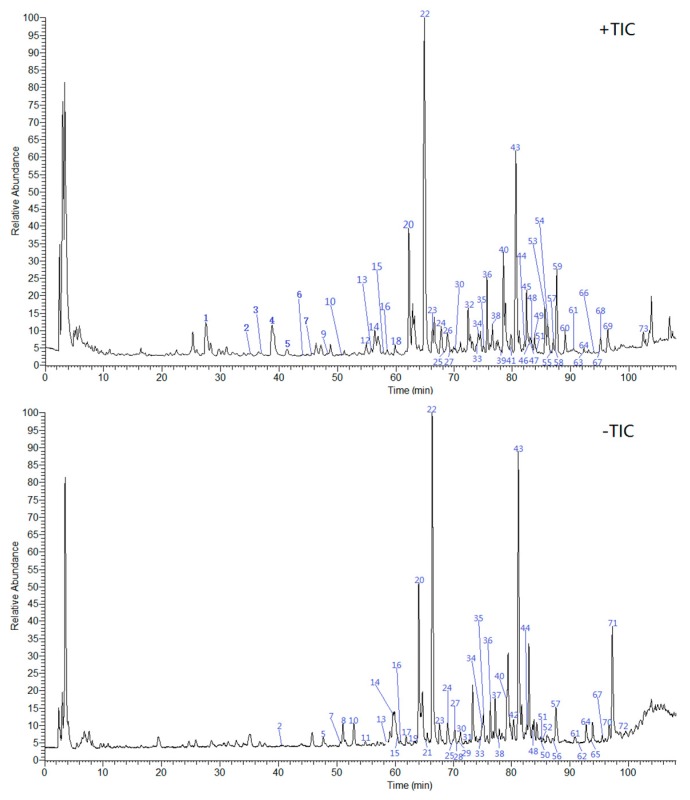
Total ion current (TIC) chromatograms of HWD. (+TIC: positive mode; −TIC: negative mode).

Total ion current (TIC) chromatograms were obtained by using UHPLC-ESI-LTQ-Orbitrap-MS (Figure 2). First, chemical constituents of seven TCMs were systematically collated and analyzed through literature retrieval. Next, the structures of compounds were searched for in authoritative chemical databases based on inferred chemical formulas or molecular weights (e.g., NIST Chemistry WebBook https://webbook.nist.gov/chemistry/ and PubChem https://pubchem.ncbi.nlm.nih.gov/). Finally, the names and structures of the compounds were preliminarily or explicitly inferred by comparing the retention time in the chromatograms and MS ion fragments with reference standards, relevant literature, and databases (e.g., Metlin https://metlin.scrips.edu/landing_page.php/pgcontent=mainpage).

**Figure 3:**
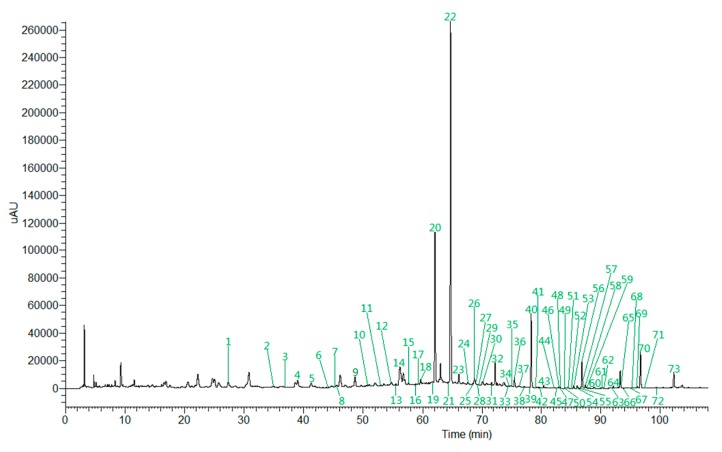
UHPLC chromatogram profiles of HWD.

A total of 73 constituents were identified. These corresponding peaks in the HPLC chromatogram profiles were labeled and are shown in Figure 3, and their chemical structures are shown in Figure S1. Seventy-three constituents can be divided into five categories: alkaloids, phenylpropanoids, flavonoids, triterpenes, and others. Among them, there are nine alkaloids (peaks 1, 3, 4, 6, 12, 18, 33, 39), 18 phenylpropanoids (peaks 8, 11, 19, 21, 28, 37, 50, 52, 56, 58, 65, 70, 71), 39 flavonoids (peaks 2, 5, 7, 9, 10, 13, 14, 15, 16, 17, 20, 22, 23, 24, 25, 26, 27, 29, 30, 31, 32, 36, 40, 41, 46, 47, 53, 57, 59, 60, 61, 62, 63, 64, 66, 67), 15 triterpenoids (peaks 34, 35, 38, 42, 43, 44, 45, 48, 49), and seven others (peaks 51, 54, 55, 68, 69, 72, 73). The peak at 73 is ketones; 72 is fatty acids; 51, 68, and 69 are lactones; and 54 and 55 are aromatic hydrocarbons. According to the elution sequence, the detailed information of the 73 chemical constituents identified in the HWDs is shown in Table S2.

After analyzing the constituents identified in UHPLC–ESI–LTQ–Orbitrap-MS, 30 CCs were identified: peaks 1, 3, 4, 6, 14, 15, 18, 20, 21, 22, 23, 24, 25, 26, 30, 31, 32, 36, 40, 43, 53, 57, 58, 60, 64, 65, 68, 69, 71, and 73. These components were further studied in network pharmacology with three common diseases (GP, FD, and CG) treated by HWDs to obtain potential quality control chemical markers as representatives to evaluate and control the quality of HWDs.

When components were identified by UHPLC-ESI-LTQ-Orbitrap-MS, it was found that peaks 3, 12, 29, 58, and 87 were rarely reported in the existing literature, and no detailed MS fragmentation patterns were studied. In this paper, as representatives of these kinds of components, these five constituents were investigated in detail with MS fragmentation methods.

#### 2.5.1. Identification of Alkaloids

Nitrogen-containing alkaline compounds were more sensitive in the positive ion mode; the hydrogen ion is easily added to R–NH_3_^+^, R_2_–NH_2_^+^, R_3_–NH^+^, R_4_–N^+^, R_3_–N, etc. to form positive charge ions [32]. Eight alkaloids were detected in HWDs in positive ion mode. Peaks 1, 3, 4, 6, 12, and 18 were from *Magnolia officinalis Cortex* and were part of the isoquinoline alkaloids, among them, peaks 1, 3, and 6 were benzylisoquinoline alkaloids and peaks 4, 12, and 18 were aporphine alkaloids. Peaks 33 and 39 were from dried tangerine peel and belong to the macrocyclic alkaloids.

**Figure 4:**
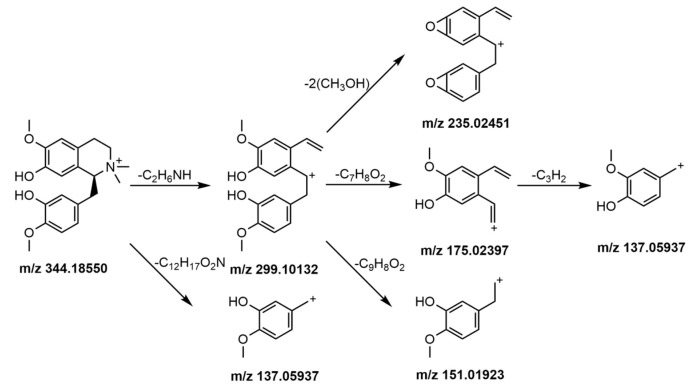
Mass spectrometric fragmentation processes of peak 3 tembetarine.

**Figure 5:**
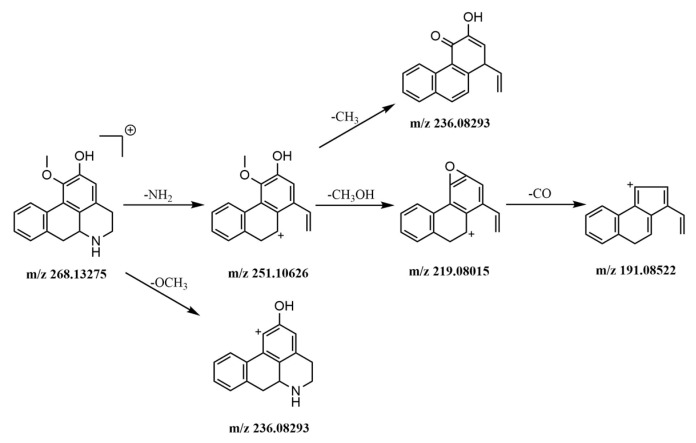
The mass spectrometric fragmentation processes of peak 12 asimilobine.

Isoquinoline alkaloids are prone to losing benzyl substituents, sometimes losing benzene ring substituents directly. It is easy to lose one molecule of methyl radical [M − (CH_3_)2NH − CH_3_]^+^ when a methoxy group is attached to a benzene ring; when there are two methyl substituents on the nitrogen atom, dimethylamine is easily lost [M − (CH_3_)2NH]^+^, or when the adjacent C on the benzene ring has methoxy and hydroxyl groups, it is easy to lose hydroxymethyl [M − (CH_3_)2NH − CH_3_OH]^+^ to obtain fragment ions. Sometimes one molecular carbonyl is further lost [M − (CH_3_)2NH − CH_3_OH – CO]^+^. As an example, the mass spectrometric fragmentation processes of peak 3 tembetarine and peak 12 asimilobine are shown in the Figure 4 and Figure 5.

#### 2.5.2. Identification of Phenylpropanoids

The seventeen phenylpropanoids identified in this study were divided into two categories: one coumarin and sixteen lignans. Peak 58 of licorice was detected in positive ion mode. Lignans are natural compounds which are synthesized from two C6–C3 units. In this study, sixteen lignans in HWDs were identified in negative ion mode, and they were all from *Magnolia officinalis Cortex*.

**Figure 6:**
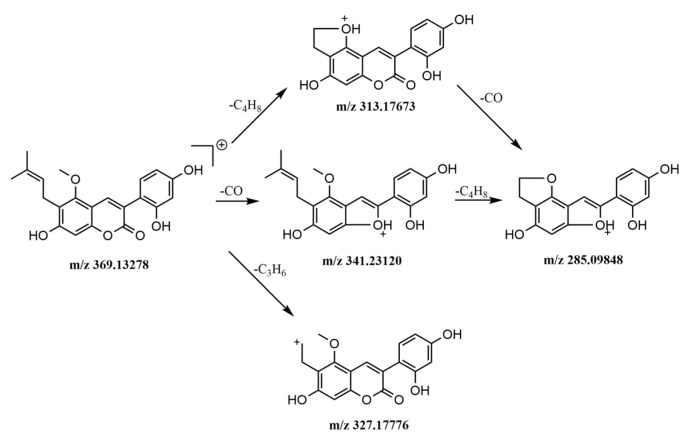
The mass spectrometric fragmentation processes of peak 58 glycycoumarin.

The protonated molecular ion *m*/*z* 369.13278 [M + H]^+^ of peak 58 was easily detected, then it lost one molecule of carbonyl to form a [M + H − CO]^+^ fragment ion of *m*/*z* 341, then further lost the branch C_4_H_8_ to form a closed loop fragment ion of m/z 285 [M + H − CO − C_4_H_8_]^+^, or the process was reversed and ion fragment *m*/*z* 313 [M + H − C_4_H_8_]^+^ occurred first and then *m*/*z* 285 [M + H − C_4_H_8_ − CO]^+^. Fragment ion *m*/*z* 327 [M + H − C_3_H_6_]^+^ was produced from *m*/*z* 369.13278 [M + H]^+^ by losing C_3_H_6_. Therefore, compared with the relevant literature, the peak 58 was determined to be glycycoumarin. The fragmentation pathways are shown in the Figure 6.

#### 2.5.3. Identification of Flavonoids

Flavonoids generally refer to the framework C_6_-C_3_-C_6_ that are formed when two phenyl rings (A and B) bind with C_3_; most of them undergo Retro Diels-Alder (RDA) cleavage. A total of thirty-six flavonoids were identified in the prescription. Three ingredients came from alpinia katsumadai (peaks 40, 57, 64), twenty ingredients came from dried tangerine peel (peaks 2, 5, 7, 9, 15, 16, 17, 20, 22, 23, 32, 36, 41, 46, 47, 53, 59, 60, 63, 66), and thirteen ingredients came from licorice (peaks 10, 13, 14, 24, 25, 26, 27, 29, 30, 31, 61, 62, 67).

**Figure 7:**
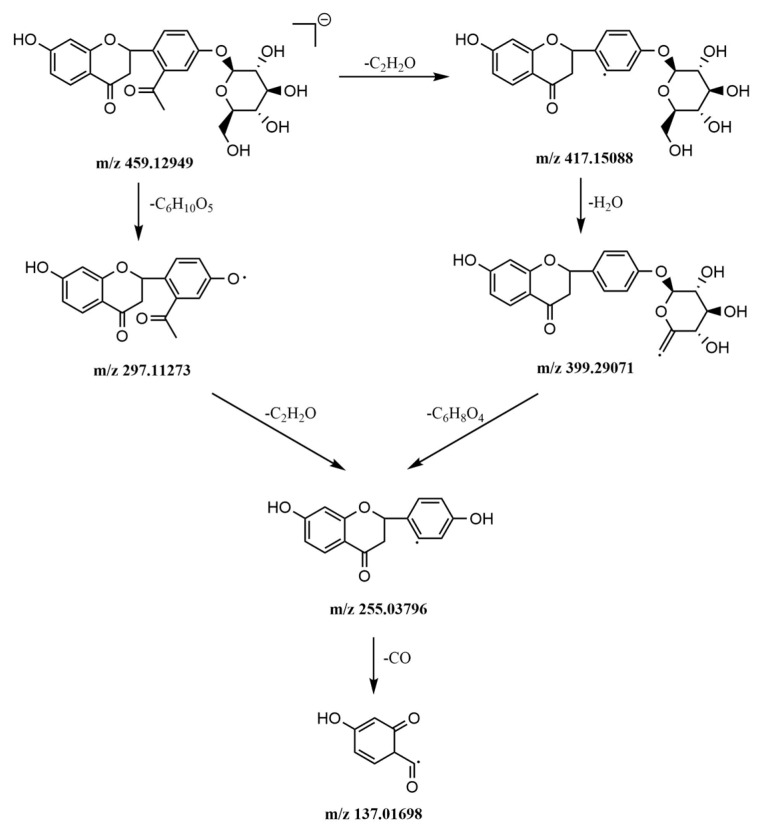
The mass spectrometric fragmentation processes of peak 29 6′-Acetyl liquiritin.

The protonated molecular ion of peak 29 was *m*/*z* 459.12949 [M − H]^−^ in negative mode, and it could form the fragment ion *m*/*z* 417 [M − H − C_2_H_2_O]^−^ by losing one acetyl group (C_2_H_2_O) at the C6′. Then, it dehydrated to form the [M − H − C_2_H_2_O − H_2_O]^−^ fragment ion of *m*/*z* 399, then form the fragment ion *m*/*z* 255 [M − H − C_2_H_2_O − H_2_O − C_6_H_8_O_4_]^−^ by losing C_6_H_8_O_4_ from the C4′, finally it decarbonylated to form the [M − H − C_2_H_2_O − H_2_O − C_6_H_8_O_4_ − CO]^−^ fragment ion of *m*/*z* 137 because of an RDA cleavage. Another pattern of fragmentation is parent ion 459.12949 [M − H]^−^ losing C_6_H_10_O_4_ and an acetyl group successively to form fragment ions *m*/*z* 297 [M − H − C_6_H_10_O_5_]^−^ and 255 [M − H − C_6_H_10_O_5_ − C_2_H_2_O]^−^. Peak 29 was assigned as 6′-Acetyl liquiritin and the fragmentation pathways are shown in the Figure 7.

#### 2.5.4. Identification of Other Compounds

Seven components were also identified in this study: peak 63 was a ketone from cardamom; peak 72 was a fatty acid from *magnolia officinalis Cortex*; peak 51 came from orange peel, peaks 68 and 69 came from auckland, they were all lactones; peak 54 came from alpinia katsumadai and peak 55 came from dried ginger, which are both aromatic hydrocarbons. The α-cleavage and Mclafferty rearrangement of γH occurs frequently in ketones.

**Figure 8:**
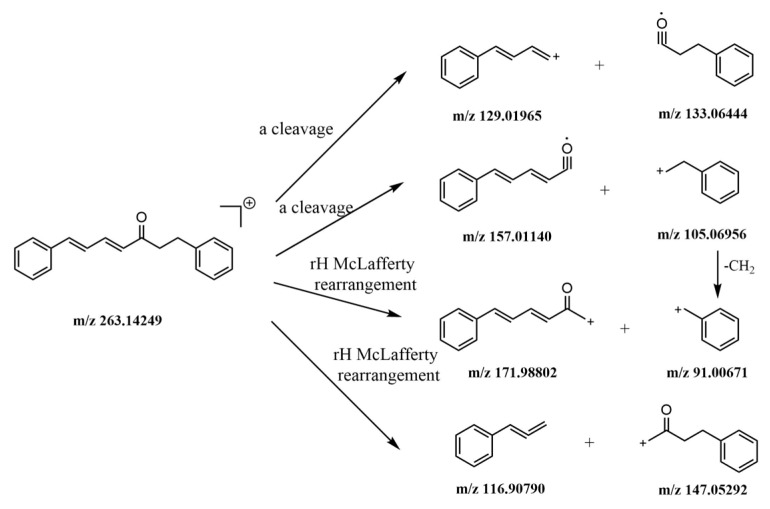
The mass spectrometric fragmentation processes of peak 73 alnustone.

As depicted in Figure 8, protonated molecular ion *m*/*z* 263.14249 [M + H]^+^ of peak 73 was observed in the positive mode. This could easily form the fragment ions *m*/*z* 129, 133, 157, and 105 after undergoing α-cleavage. Meanwhile, fragment ions *m*/*z* 147, 116, 171, and 91 were produced by Mclafferty rearrangement of γH. After comparing the reference substances, the peak 73 was identified as alnustone.

### 2.6. Identification of Twelve Chemical Ingredients by Comparison with Reference Substances

**Figure 9:**
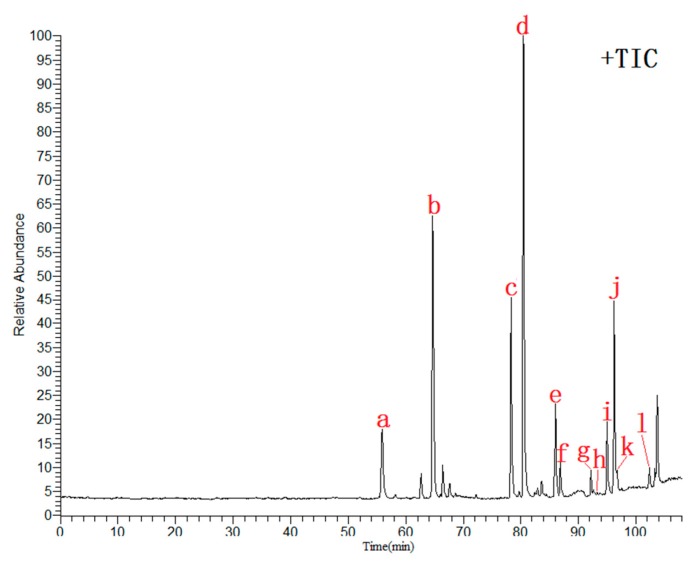
Total ion chromatogram of the reference substances in positive ion mode (a. Liquiritin; b. Hesperidin; c. Alpinetin; d. Glycyrrhizic acid; e. 6-Gingerol; f. Pinocembrin; g. Cardamonin; h. Honokiol; i. Costunolide; j. Dehydrocostuslactone; k. Magnolol; l. Alnustone).

In order to accurately identify more ingredients in the prescription, twelve common peaks that appeared steadily in fifteen batches were calibrated, and the samples were compared with the reference substances. Twelve components were identified: liquiritin (peak a), hesperidin (peak b), alpinetin (peak c), glycyrrhizic acid (peak d), pinocembrin (peak e), cardamonin (peak f), 6-gingerol (peak g), honokiol (peak h), costunolide (peak i), dehydrocostuslactone (peak g), magnolol (peak k), and alnustone (peak l). HPLC chromatography and mass spectrometry total ion current (TIC) chromatograms are shown in Figure 9.

### 2.7. Network Pharmacology

#### 2.7.1. cCCs-cT Network

**Figure 10:**
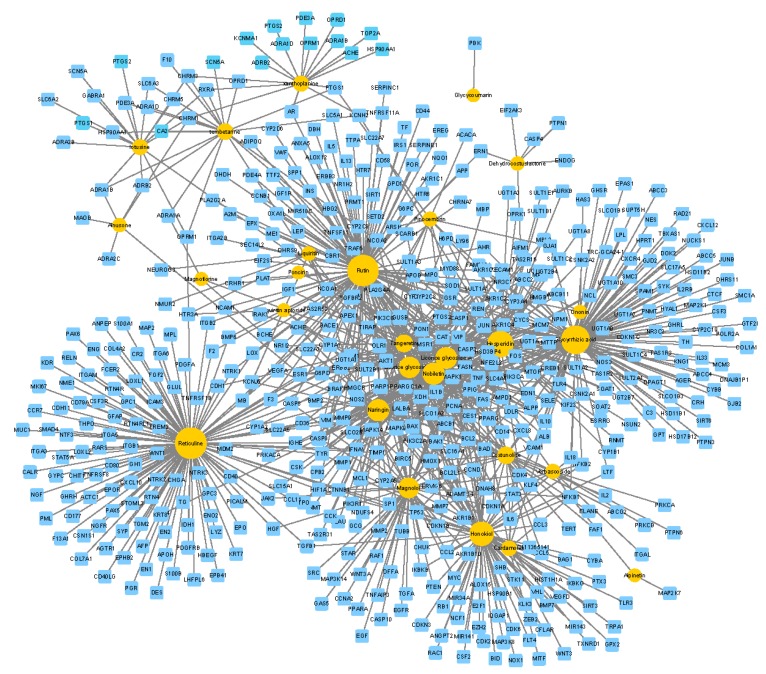
Network of 27 candidate common components (CCs) (orange) predicted to have 539 candidate targets (sky blue).

Based on our prediction, the cCCs-cT network (Figure 10) included 566 nodes (27 candidate CCs and 539 candidate targets) and 977 edges. The circle size of the CCs in the network increases with the number of edges (degree of targets). From the figure, we can see that individual components have only one target, while the other components have multi-target characteristics, which may correspond to various pathways and mechanisms for the treatment of diseases.

#### 2.7.2. GP/FD/CG-cT Network

**Figure 11:**
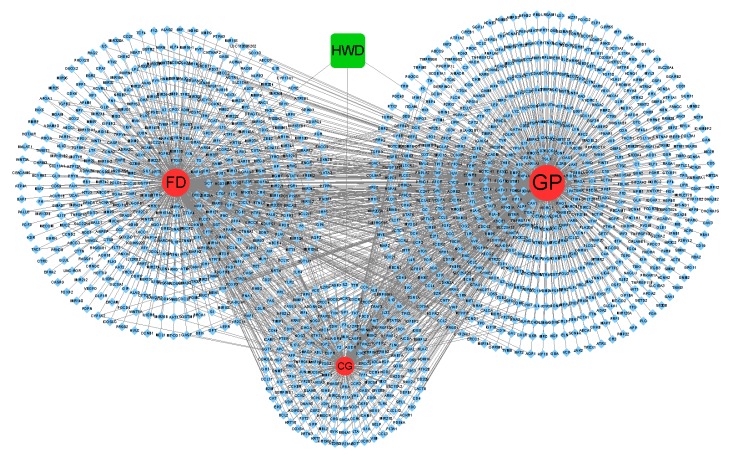
Network of three diseases, gastric pain (GP), chronic gastritis (CG), and functional dyspepsia (FD) (red), predicted to have 1284 candidate targets (blue).

As shown in Figure 11, the GP/FD/CG-cT network comprised 1288 nodes (3 diseases, 1 HWD, 1284 candidate targets, and 2050 edges). Nine hundred eighty-five candidate targets of GP, 692 of FD, and 370 of CG were obtained. In order to make the results more intuitive, the size of the circle (3 diseases) also changes with the number of edges (degree of targets). We can see that GP links with the most targets, CG links the least targets, and some targets of the three diseases intersect with each other.

#### 2.7.3. pCCs-pT-GP/FD/CG Network

**Figure 12:**
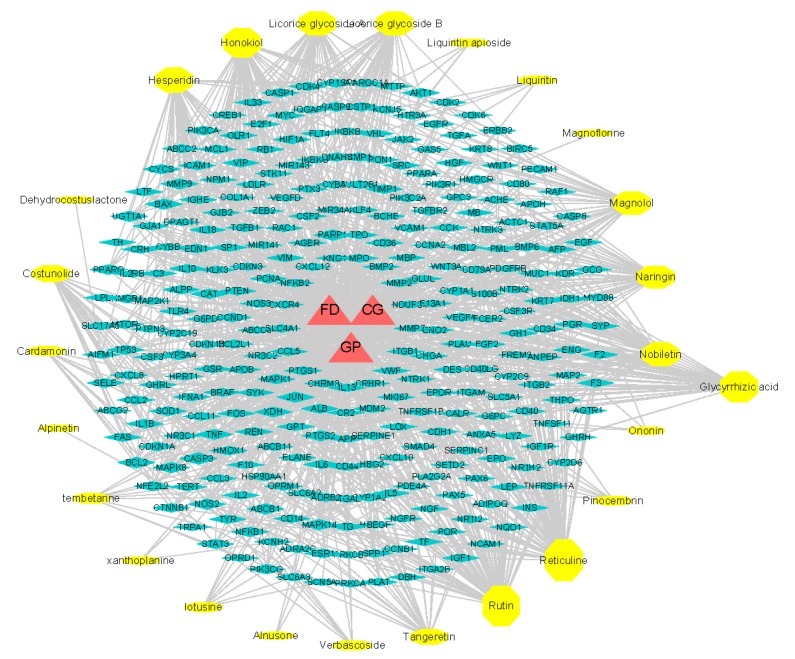
Network of three diseases GP, CG, FD (red) and 25 potential active ingredients (yellow) predicted to have 303 candidate targets (blue).

**Figure 13:**
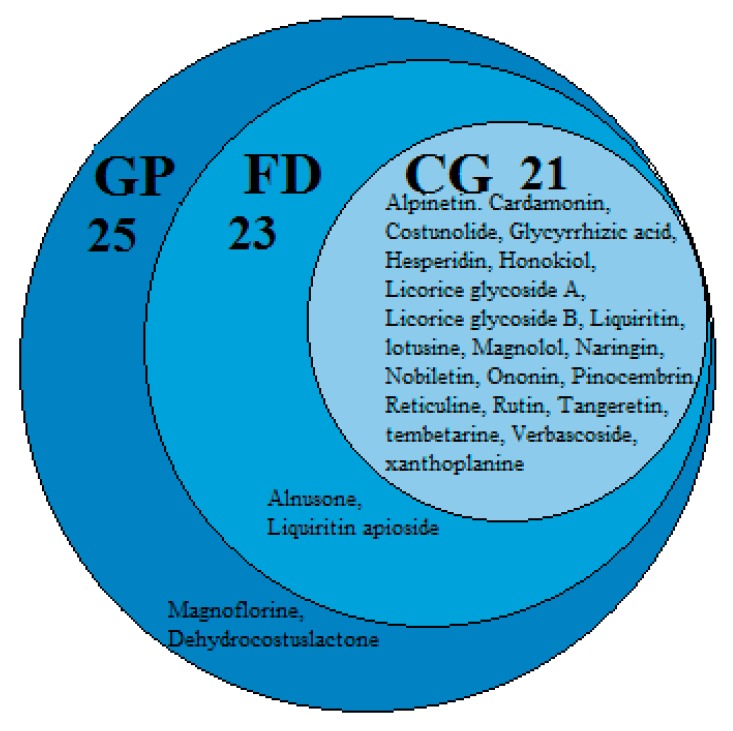
Distribution of 25 potential active ingredients in three diseases.

To further find the potential active ingredient markers, the pCCs-pT-GP/FD/CG network consisted of 331 nodes (3 diseases, 25 potential active ingredients, 303 candidate targets, and 1305 edges). The size of all nodes varied with the number of edges (degree of targets) they connect (Figure 12). Comparing the results, we found 303 potential common targets of 25 potential active ingredients with GP/FD/CG. Twenty-five potential active ingredients had 264 potential common targets with GP, 23 potential active ingredients had 218 potential common targets with FD, and 21 potential active ingredients had 162 potential common targets with CG (Figure 13).

In order to screen potential quality control markers in combination with pharmacology, four of 21 common potential active ingredients of the three diseases were selected as potential active ingredient markers of HWDs based on the degree of target, professional knowledge, and literature validation.

### 2.8. Establishment of the Method of Quantitative Determination by HPLC

#### 2.8.1. Linearity Range, Limit of Detection (LOD), and Limit of Quantitation (LOQ)

The mixed standard reserve solution containing four potential active ingredient markers was diluted into six appropriate concentrations and each concentration was tested three times. Calibration curves were established by peak areas and concentrations of standard solutions. The LOD and LOQ under current conditions were determined at the signal-to-noise ratios of 3:1 and 10:1, respectively. Detailed information on the calibration curve range, LOD and LOQ are shown in Table 1. The results showed that there was a good linear relationship between the concentrations of the four compounds and their peak areas within the test range (r^2^ > 0.9997).

#### 2.8.2. Precision, Reproducibility, Stability, and Recovery

The instrument’s intra-day and inter-day precision were evaluated by six consecutive measurements of the same mixed standard solution in one day and three consecutive measurements of the same mixed standard solution in three consecutive days. Continuous determination of six samples was prepared by the same method to assess the repeatability of the method. The same sample was tested at 0, 2, 4, 6, 8, 10, 12, and 24 h to evaluate the stability of the sample. The recovery test was determined by adding 80%, 100%, and 120% mixed reference standards of the sample content to HWD samples. As shown in Table 2, the relative standard deviation (RSD) values of precision, reproducibility, stability, and recovery for the four potential active ingredient markers were all less than 3%, and the recoveries of the four potential active ingredient markers ranged from 100.33% to 102.49%, which means that the quantitative determination method for simultaneous determination of the four potential active ingredient markers is feasible and reliable.

### 2.9. Quantitative Determination of Four Potential Active Ingredient Markers in 15 Batches of HWD

In order to further test the practicability and universality of the above quantitative determination method, the contents of the four potential active ingredient markers in 15 batches of HWD samples were determined (Table 3). The results show that this method can be used for the quantitative determination of HWDs conveniently, quickly, and scientifically, and can be used as a means to evaluate and control the quality of HWDs so as to ensure the stability and uniformity of clinical therapeutic effect.

## 3. Discussion

An HPLC fingerprint method of HWDs was established for the first time through 15 batches of samples. As a means of quality evaluation and control of HWDs, it has the characteristics of convenience, rapidity, and comprehensiveness. As reported, polysaccharides from different parts or species of ganoderma or polysaccharides from the same parts of ganoderma but from different geographical regions or different strains can be differentiated clearly by HPLC fingerprint [33]. Therefore, the fingerprint method has become a widely recognized and important method for quality evaluation of TCM. Similarity analysis is an important helper for fingerprints to play a better role [34]. In this study, 15 batches of fingerprints were imported into similarity software to generate a simulated reference fingerprint of HWDs, and 45 CCs were selected as common characteristic components of HWDs from different origins. The elution time of the 45 CCs ranged from 7.065 min to 101.903 min, including a variety of compounds ranging from small polarity to large polarity.

The chemical constituents in TCM are numerous and complex, but multi-batch CCs may be a breakthrough to further explain the therapeutic effect and mechanism of HWDs. In this paper, 73 constituents were identified for the first time by UHPLC coupled with ESI-LTQ-Orbitrap-MS in both negative and positive modes. Thirty of 45 CCs in 15 batches of HWDs were identified, 12 of which were confirmed by comparison with reference substances. This lays a foundation for further research on the potential active ingredients and potential targets of HWDs. As a more mature method for chemical identification and analysis, UHPLC-ESI-LTQ-Orbitrap-MS has increasingly become a powerful technical support for the study of complex systems of TCM [35].

TCM prescriptions often play a significant role in clinical treatment, but the effective component group is a difficult problem in the mechanism and quality control of prescriptions [36]. Drug–target and target–disease networks constructed by network pharmacology can be used to identify active ingredients and potential targets [37]. Weiyang Tao et al. [38] and Tang F et al. [39] predicted 58 and 41 active ingredients, respectively, that have potential targets with related diseases from Radix Curcumae formula and Mahuang Fuzi Xixin decoction based on network pharmacology. This study successfully constructed cCCs-cT, GP/FD/CG-cT, and GP/FD/CG-cT networks. Four CCs were screened as potential active ingredient markers of HWDs based on the degree of target (degree > 40), professional knowledge, and literature validation. It has been reported that magnolol, honokiol, and hesperidin can significantly increase gastrointestinal motility, improve functional dyspepsia [40,41], and relieve stomach pain [42] and glycyrrhizic acid has protective effects on gastric mucosa against gastritis [43]. These four potential active ingredient markers were eluted by HPLC at 63.539 min, 80.142 min, 92.857 min, and 96.268 min, respectively, which are likely to be the key active ingredients in HWDs for the treatment of gastric-related diseases.

Quantitative determination method was established to evaluate the quality of HWDs, and the contents of four potential active ingredient markers in 15 batches of HWDs were determined, which verified the universality and scientific basis of this method. The contents of the four components in 15 batches of HWDs were different. The content ranges of hesperidin, glycyrrhizic acid, honokiol, and magnolol in the 15 batches of HWDs were 210.6112–309.1562 μg/mL, 108.1412–336.2496 μg/mL, 6.0745–84.8151 μg/mL, and 24.3066–24.3066 μg/mL, respectively. The response values of individual peaks are obviously different from those of other peaks when the overall similarity of fingerprints is strong. It may be related to the origin and harvesting time of TCMs, or influenced by the interaction of chemical components. Subsequent experiments will be carried out on the influence of origin, harvesting time, and other factors on the overall quality and fingerprint of HWDs.

Although the mechanism and targets of HWDs in the treatment of gastric-related diseases cannot be fully explained by the existing work, through the combination of multi-batch fingerprint, UHPLC-ESI-LTQ-Orbitrap-MS, and network pharmacology, four reliable components closely related to the potential targets of gastric-related diseases were successfully screened. This new idea of evaluating the quality of HWDs by these four potential active ingredients obtained from the above research is very advanced and reliable. It can be used as a reference for the study of pharmacodynamics and quality control of other TCM prescriptions. Follow-up experiments will further explore the effect and mechanism of the 15 batches of HWDs in the treatment of gastric-related diseases. Whether the four potential active component markers can be used as an effective component group to replace HWDs to achieve therapeutic effect will also be the focus of future research.

## 4. Materials and Methods

### 4.1. Reagents and Materials

In this study, all batches of medicinal materials of HWDs were purchased from Yifan Pharmaceutical Co. Ltd. (Zhejiang, China), which were collected in different regions of China. The origins and batch numbers of seven TCMs of 15 batches of HWD samples are shown in the Table S1. Reference standards of magnolol (110729-201714), honokiol (110730-201614), hesperidin (110721-201818), ammonium glycyrrhizinate (110731-201720), costunolide (111524-201710), dehydrocostuslactone (111525-201711), liquiritin (111610-201607), alpinetin (110762-201806), pinocembrin (111829-201703), cardamonin (110763-201604), alnustone (111830-201703), and 6-gingerol (111833-201705) were obtained from the National Institutes for Food and Drug Control (Beijing, China). Purified water for experiments was purchased from Wahaha Corporation (Hangzhou, China). HPLC-grade acetonitrile, phosphoric acid, and methanol were acquired from Fisher Corporation (Shanghai, China). HPLC/MS-grade acetonitrile and formic acid were bought from Sigma-Aldrich Corporation (Shanghai, China). All other reagents and chemicals used were of analytical grade.

### 4.2. Preparation of Lyophilized Powder of HWD

All dry Chinese herbal pieces were first crushed into a powder and sieved (10 mesh). Four grams Magnolia officinalis Cortex, 4 g Citri Reticulatae Pericarpium, 2 g Radix aucklandiae, 2 g Glycyrrhiza uralensis Fisch, 2 g Semen Alpiniae Katsumadai, 2 g Poria cocos, and 0.3 g Rhizoma Zingiberis were accurately weighed, mixed, and boiled for 1 h with 300 mL water, then filtered. The volume of the filtrate was adjusted to 250 mL, then 5 mL of the filtrate was precisely transferred into a penicillin bottle and transformed into lyophilized powder in a freeze dryer at −80 °C.

### 4.3. Preparation of Sample Solutions for Fingerprint Analysis and UHPLC-ESI-LTQ-Orbitrap-MS Analysis

First, 5 mL 70% methanol aqueous solution was precisely transferred into a penicillin bottle containing lyophilized powder, then the sample were extracted by ultrasound (KQ5200DA, 250 W, 40 kHz, Kunshan, China) for 15 min and shaken well. Subsequently, the extract was filtered through a 0.45 μm microporous membrane to prepare the tested samples.

### 4.4. Preparation of Standard Solutions

Magnolol, honokiol, hesperidin, and ammonium glycyrrhizinate were accurately weighed in brown volumetric flasks, and reserve solutions with methanol were prepared separately. A series of concentrations in the ranges of 3.626–14.473 μg/mL to 259.00–530.18 μg/mL of standard mixtures were prepared by diluting the individual reserve standard solution with methanol. All standard solutions were stored at 4 °C before use.

### 4.5. Instrumentation and Chromatographic Conditions

#### 4.5.1. HPLC Conditions for Fingerprint Analysis

Fingerprint analysis and quantitative analysis were conducted on a Dionex Utimate 3000 Series HPLC system (Thermo Fisher, Shanghai, China) equipped with a quaternary solvent delivery system, an ultraviolet detector with full wavelength scanning, and a column temperature controller.

All samples of fingerprint analysis and quantitative analysis were analyzed at a column temperature of 30 °C on a Thermo Scientific-C18 column (4.6 × 250 mm, 5 μm, Thermo Fisher, Shanghai, China). The mobile phase consisted of 0.1% phosphoric acid aqueous solution (eluent A) and acetonitrile (eluent B), the gradient elution mode was set as follows: 2–7% B at 0–6 min, 7–9% B at 6–10 min, 9% B at 10–15 min, 9–13% B at 15–35 min, 13% B at 35–40 min, 13–19% B at 40–54 min, 19–29% B at 54–66 min, 29–80% B at 66–103 min, and 80% B at 103–108 min. The injection volume was 10 μL and the flow rate was 1.0 mL/min.

#### 4.5.2. Conditions for UHPLC-ESI-LTQ-Orbitrap-MS Analysis

MS detection was carried out with a mass spectrometer (Thermo Scientific, LTQ-Orbitrap XL, Shanghai, China). The mass spectrometer was operated both in positive and negative ion mode. The operation conditions were as follows: capillary temperature, 350 °C; apci vaporizer temperature, 300 °C; source voltage, 4 kV; aux gas flow, 10 L/min; capillary voltage, 25 V; sheath gas flow, 30 L/min; and Tube Lens, 110 V (positive ion mode) and −110 V (negative ion mode). The scan range of the mass spectra was 100–1500 *m/z*. System control, high speed data acquisition, real-time data display, and data processing were accomplished by Thermo Workstation Acquisition Software Xcalibur Version 2.2 (Thermo Fisher Scientific, Waltham, MA, USA) and UHPLC analysis Software Chromeleon Version 7.0 (Thermo Fisher Scientific, Waltham, MA, USA)

The chromatographic conditions for UHPLC-ESI-LTQ-Orbitrap-MS analysis were the same as those for the fingerprint analysis and quantitative analysis except that 0.1% formic acid replaced 0.1% phosphoric acid.

### 4.6. Similarity Analysis (SA)

Similarity analysis was progressed by Similarity Evaluation System (SES) for Chromatographic Fingerprint of TCM software (Version 2012, Chinese Pharmacopoeia Committee, Beijing, China). It calculated and generated simulated reference fingerprints, and counted separately the correlation coefficients of similarity between 15 batches of HWDs and simulated reference fingerprints.

### 4.7. Network Pharmacology (NP)

The candidate targets of CCs and three diseases (GP, FD, and CG) commonly treated by HWDs were obtained from the TCMSP database (http://lsp.nwu.edu.cn/tcmsp.php) and Genecards database (http://www.genecards.org/). Network constructions were made by Cytoscape software (Version 3.7.1, Cytoscape Consortium, Bethesda, MD, USA) (http://www.cytoscape.org/).

We established networks as follows:

(1) the ‘candidate CCs-candidate target (cCCs-cT) network’ was established by linking candidate CCs with all of their candidate targets;
(2) the ‘GP, FD, CG-candidate target (GP/FD/CG-cT) network’ was established by inking GP, FD, and CG with all of their candidate targets;
(3) the ‘potential CCs-potential target-GP, FD, CG (pCCs-pT-GP/FD/CG) network’ was constructed by connecting potential CCs and GP, FD, and CG with their potential targets, which were used to screen out potential active ingredient markers.

If the correlation of candidate targets is low (relevance score < 5), the candidate targets are excluded. On the basis of degree of targets (degree > 40) and professional knowledge, the screened ingredients were compared with the reported literature to confirm the final potential active ingredients.

## 5. Conclusions

HWDs have had a remarkable therapeutic effect on GP, FD, and CG in clinical treatment for hundreds of years; the chemical constituents of HWDs are numerous and complex. It is necessary to find potential active ingredients related to therapeutic effect to evaluate the quality of HWDs. Based on the combined application of three mature and reliable technologies, a new idea for quality control of HWDs was put forward in this paper. Firstly, the fingerprint method of HWDs was established. Forty-five CCs were screened out by 15 batches of fingerprints. Then, 73 chemical constituents were identified in HWDs by UHPLC-ESI-LTQ-Orbitrap-MS, and 30 CCs were identified. In addition, the mass spectrometric decomposition of some components, which have not been reported in detail in the literature, were speculated. Next, the candidate targets of 27 CCs and three diseases (GP, FD, and CG) were obtained from databases. After eliminating low correlative targets, the networks of candidate CCs, GP, FD, CG, and candidate targets were successfully constructed by network pharmacology. Four CCs were screened as potential active ingredient markers of HWDs based on the degree of target (degree > 40), professional knowledge, and literature validation. Finally, quantitative determination of the four components in HWDs was established and the quality of 15 batches of HWDs was determined successfully. A rapid, reasonable, and effective method for quality evaluation and control of HWDs was established. Taking HWDs as an example, it is a new idea to use fingerprints, UHPLC-ESI-LTQ-Orbitrap-MS, and network pharmacology to screen CCs as potential quality control indicators to evaluate and control the quality of prescriptions. This is the basis for further development and research of HWDs, and provides a new way of thinking for the research of other TCM prescriptions.

## Figures and Tables

**Table 1:** Calibration curve, limit of detection (LOD) and limit of quantitation (LOQ) of four potential active ingredient markers (*n* = 6).

Analyte	Linear Range (μg/mL)	Calibration Curve	r^2^	LOD (μg/mL)	LOQ (μg/mL)
Magnolol	10.485–262.13	y = 0.7850x + 0.9241	0.9999	1.02	3.78
Honokiol	3.626–259.00	y = 0.5289x + 0.5954	0.9999	0.738	2.61
Hesperidin	10.752–530.18	y = 0.3495x − 0.4054	0.9999	0.582	1.23
Glycyrrhizic acid	14.473–347.68	y = 0.0609x − 0.8267	0.9997	1.15	3.14

**Table 2:** Precision, repeatability, stability, and recovery results of four potential active ingredient markers. RSD = relative standard deviation.

Analyte	Precision		Reproducibility (*n* = 6)	Stability (*n* = 10)	Recovery (*n*= 3)	
	Intra-day (*n* = 6)	Inter-day (*n* = 3)				
	RSD (%)	RSD (%)	RSD (%)	RSD (%)	Mean	RSD (%)
Magnolol	0.94	1.2	1.95	1.06	101.93	1.95
Honokiol	1.12	1.34	2.03	1.26	100.75	0.46
Hesperidin	0.89	1.03	1.47	1.15	100.33	0.79
Glycyrrhizic acid	0.57	0.89	2.42	0.97	102.49	0.55

**Table 3:** The contents of four analytes and similarity values for 15 batches of HWDs (μg/mL, Mean ± SD, *n* = 3).

Sample	Hesperidin	Glycyrrhizic Acid	Honokiol	Magnolol	Similarity Value
S1	278.2418 ± 0.0182	205.9195 ± 0.0212	43.6655 ± 0.0214	33.9597 ± 0.0340	0.978
S2	268.8197 ± 0.0049	162.8062 ± 0.0022	16.5347 ± 0.0130	24.3066 ± 0.0187	0.946
S3	292.2355 ± 0.0006	127.5041 ± 0.0025	84.8151 ± 0.0005	32.8499 ± 0.0057	0.962
S4	309.1562 ± 0.0013	109.7176 ± 0.0062	71.0671 ± 0.0092	40.5120 ± 0.0107	0.980
S5	231.4275 ± 0.0275	189.0328 ± 0.0267	63.9387 ± 0.0106	36.3183 ± 0.0174	0.976
S6	210.6112 ± 0.0229	138.1133 ± 0.0379	36.3562 ± 0.0018	33.0920 ± 0.0041	0.970
S7	236.1765 ± 0.0315	145.0608 ± 0.0420	73.6863 ± 0.0087	58.3732 ± 0.0061	0.960
S8	246.5579 ± 0.0050	336.2496 ± 0.0006	23.8629 ± 0.0106	38.3361 ± 0.0123	0.930
S9	247.4581 ± 0.0060	127.2972 ± 0.0073	32.3751 ± 0.0038	27.9307 ± 0.0007	0.968
S10	248.4521 ± 0.0095	150.5337 ± 0.0084	64.5708 ± 0.0209	44.1939 ± 0.0295	0.964
S11	293.0197 ± 0.0328	131.7915 ± 0.0474	72.2159 ± 0.0289	55.7920 ± 0.0409	0.956
S12	273.9662 ± 0.0052	181.4631 ± 0.0109	10.4443 ± 0.0249	31.1806 ± 0.0333	0.946
S13	281.9760 ± 0.0164	138.7783 ± 0.0275	6.0745 ± 0.0061	43.7450 ± 0.0046	0.922
S14	284.3605 ± 0.0151	113.6962 ± 0.0188	78.9074 ± 0.0251	29.3832 ± 0.0385	0.973
S15	282.6103 ± 0.0034	108.1412 ± 0.0037	51.4084 ± 0.0162	48.8744 ± 0.0273	0.957

## Supplementary Materials

The following are available online. Table S1: Sources and batch numbers of TCM in fifteen batches of HWD samples; Table S2: Detailed information on 88 chemical constituents of HWDs and seven drugs. Figure S1: Structural formulas of 73 chemical constituents.
