# Supplementary material for: Study on the Material Basis of Houpo Wenzhong Decoction by HPLC Fingerprint, UHPLC-ESI-LTQ-Orbitrap-MS, and Network Pharmacology
Source: Molecules. 2019 Jul 14;24(14):2561. doi: 10.3390/molecules24142561 (PMC6681077; doi:10.3390/molecules24142561)
Supplement: Supplementary file 1 [file molecules-24-02561-s001.pdf]

Supplementary Materials

Study on the Material Basis of Houpo Wenzhong Decoction by HPLC fingerprint, UHPLC-ESI-LTQ-Orbitrap-MS and Network Pharmacology

Juyuan Luo[1], Gongsen Chen[1], Donghan Liu[1], Yan Wang[1], Qi Qi[1], Haiyan Hu[1], Pengyue Li[1], Jie Bai[1,*], Shouying Du[1,*], Yang Lu[1,*], Yuming Wang[2] and Cun Liu[2]

[1] School of Chinese Materia Medica, Beijing University of Chinese Medicine, Yangguang South Avenue, Fangshan District, Beijing 102488, China; luojy0329@163.com (J.L.); gongsenchen@163.com (G.C.); liudonghan80@163.com (D.L.); wy18713922886@163.com (Y.W.); doubleseven776@163.com (Q.Q.);

yxyhhy@163.com (H.H.); pengyuelee@126.com (P.L.);

[2] Yifan Pharmaceutical Co.,Ltd, Lin an, Zhejiang 311300, China; wangyuming@yifanyy.com (Y.W.); liucun@yifanyy.com (C.L.)

* Correspondence: baijie22811@163.com (J.B.); dusy@bucm.edu.cn (S.D.); landocean28@163.com (Y.L.); Tel.: +86-010-8473-8657 (J.B.), +86-010-8473-8615 (S.D.), +86-010-5391-2123 (Y.L.).

Table S1. Sources and batch numbers of TCM in fifteen batches of HWD samples.

SN	*Magnolia officinalis Cortex*		*Citri Reticulatae Pericarpium*		*Radix aucklandiae*		*Semen Alpiniae Katsumadai*		*Glycyrrhiza uralensis Fisch*		*Poria cocos*		*Rhizoma Zingiberis*	
	**BN**	**Origins**	**BN**	**Origins**	**BN**	**Origins**	**BN**	**Origins**	**BN**	**Origins**	**BN**	**Origins**	**BN**	**Origins**
S1	HP-14	Enshi, Hubei	CP-2	Yibin, Sichuan	MX-2	Lijiang, Yunnan	CDK-3	Haikou, Hainan	GC-10	Ordos, Inner Mongolia	FL-3	Shangluo, Shanxi	GJ-2	Qianwei, Sichuan
S2	HP-11	Enshi, Hubei	CP-2	Yibin, Sichuan	MX-3	Lijiang, Yunnan	CDK-2	Cenxi, Guangxi	GC-13	Ordos, Inner Mongolia	FL-2	Yuexi, Anhui	GJ-1	Yunnan
S3	HP-13	Enshi, Hubei	CP-4	Guangdong	MX-6	Chuxiong, Yunnan	CDK-3	Haikou, Hainan	GC-2	Longxi, Gansu	FL-2	Yuexi, Anhui	GJ-2	Qianwei, Sichuan
S4	HP-4	Beichuan, Sichuan	CP-8	Chengdu, Sichuan	MX-8	Heqing, Yunnan	CDK-2	Cenxi, Guangxi	GC-3	Longxi, Gansu	FL-1	Yunnan	GJ-6	Changshun, Guizhou
S5	HP-2	Beichuan, Sichuan	CP-6	Hangzhou, Zhejiang	CP-5	Lijiang, Yunnan	CDK-3	Haikou, Hainan	GC-11	Ordos, Inner Mongolia	FL-3	Shangluo, Shanxi	GJ-2	Qianwei, Sichuan
S6	HP-8	Pingwu, Sichuan	CP-5	Longyan, Fujian	CP-5	Lijiang, Yunnan	CDK-3	Haikou, Hainan	GC-4	Longxi, Gansu	FL-3	Shangluo, Shanxi	GJ-6	Changshun, Guizhou
S7	HP-9	Pingwu, Sichuan	CP-5	Longyan, Fujian	MX-1	Lijiang, Yunnan	CDK-2	Cenxi, Guangxi	GC-12	Ordos, Inner Mongolia	FL-1	Yunnan	GJ-5	Guizhou
S8	HP-7	Pingwu, Sichuan	CP-1	Yibin, Sichuan	MX-8	Heqing, Yunnan	CDK-1	Yunnan	GC-15	Ordos, Inner Mongolia	FL-2	Yuexi, Anhui	GJ-1	Yunnan
S9	HP-3	Beichuan, Sichuan	CP-3	Yibin, Sichuan	MX-7	Chuxiong, Yunnan	CDK-1	Yunnan	GC-1	Longxi, Gansu	FL-1	Yunnan	GJ-4	Qianwei, Sichuan
S10	HP-15	Wanyuan, Sichuan	CP-6	Hangzhou, Zhejiang	MX-1	Lijiang, Yunnan	CDK-1	Yunnan	GC-5	Longxi, Gansu	FL-1	Yunnan	GJ-3	Qianwei, Sichuan
S11	HP-6	Pingwu, Sichuan	CP-7	Jiangmen, Guangdong	MX-7	Chuxiong, Yunnan	CDK-1	Yunnan	GC-9	Altay, Xinjiang	FL-1	Yunnan	GJ-4	Qianwei, Sichuan
S12	HP-12	Enshi, Hubei	CP-1	Yibin, Sichuan	MX-6	Chuxiong, Yunnan	CDK-2	Cenxi, Guangxi	GC-8	Altay, Xinjiang	FL-3	Shangluo, Shanxi	GJ-4	Qianwei, Sichuan
S13	HP-1	Beichuan, Sichuan	CP-4	Guangdong	MX-3	Lijiang, Yunnan	CDK-3	Haikou, Hainan	GC-14	Ordos, Inner Mongolia	FL-2	Yuexi, Anhui	GJ-6	Changshun, Guizhou
S14	HP-10	Enshi, Hubei	CP-8	Chengdu, Sichuan	MX-2	Lijiang, Yunnan	CDK-1	Yunnan	GC-6	Altay, Xinjiang	FL-2	Yuexi, Anhui	GJ-3	Qianwei, Sichuan

S15	HP-5	Pingwu, Sichuan	CP-7	Jiangmen, Guangdong	MX-4	Lijiang, Yunnan	CDK-2	Cenxi, Guangxi	GC-7	Altay, Xinjiang	FL-3	Shangluo, Shanxi	GJ-5	Guizhou

SN: sample number, BN: Batch number.

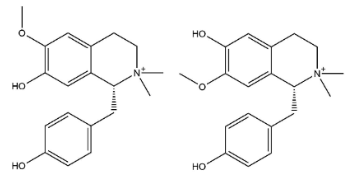

1、Magnocurarine or Lotusine

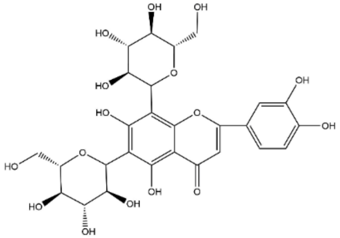

2、Luteolin-6,8-di-C-glucoside

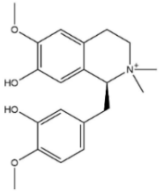

3、Tembetarine

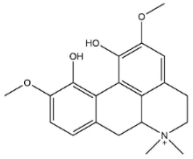

4、Magnoflorine

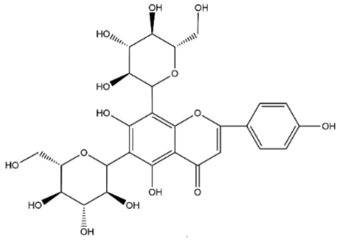

5. Vicenin-2

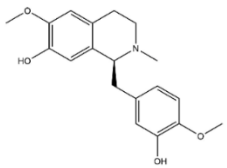

6. Reticuline

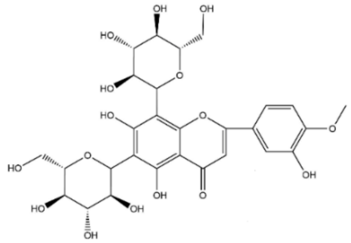

7. Diosmetin 6,8-di-C-glucoside

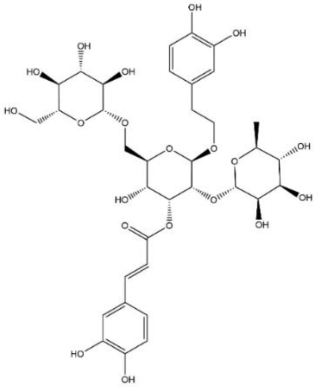

8. Magnoloside B

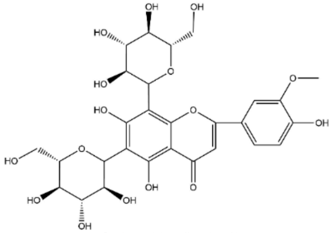

9. Chrysoeriol 6,8-di-C-glucoside

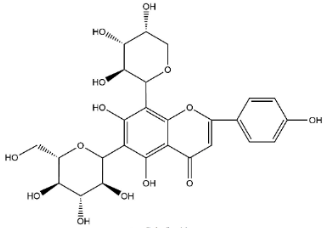

10. Schaftoside

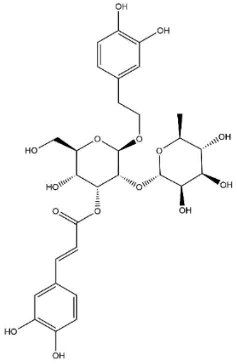

11. Magnoloside A

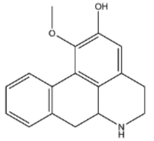

12. Asimilobine

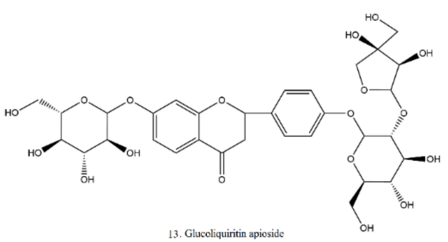

13. Glucoliquiritin apioside

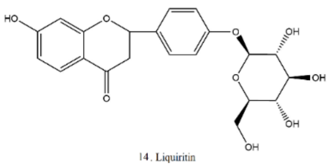

14. Liquiritin

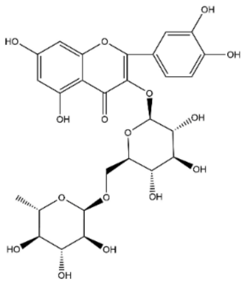

15. Rutin

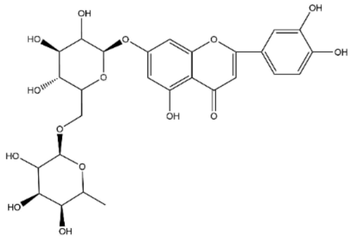

16. Luteolin-7-rutinoside

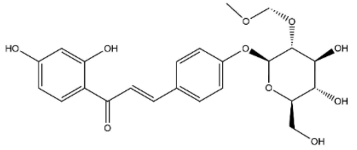

17. Eriocitrin

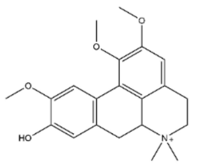

18. Xanthoplanine

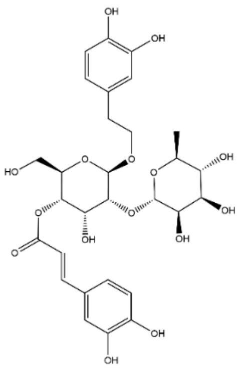

19. Magnoloside M

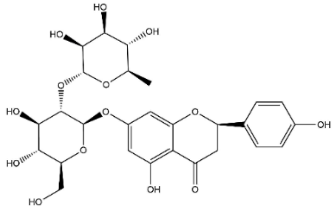

20. Naringin

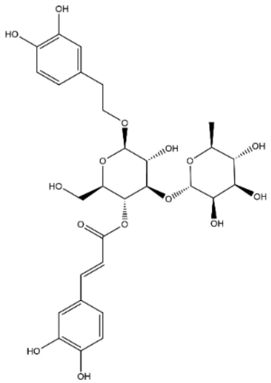

21. Verbascoside

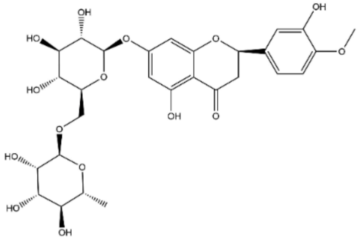

22. Hesperidin

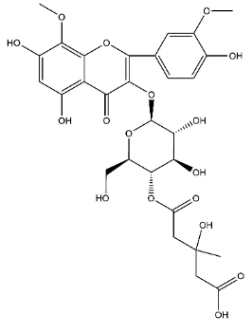

23. Limocitrin-3-O-(3-hydroxy-3-methylglut-arate)-glucoside or isomers

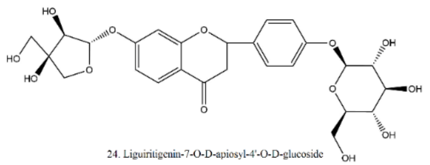

24. Liguiritigenin-7-O-D-apiosyl-4'-O-D-glucoside

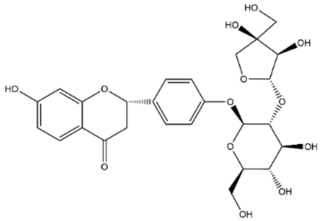

25. Liquiritin apioside

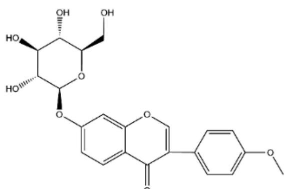

26. Ononin

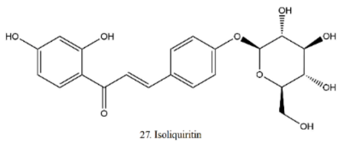

27. Isoliquiritin

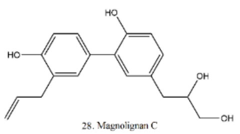

28. Magnolignan C

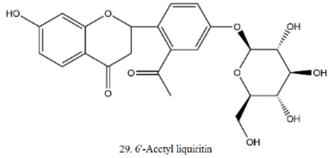
29. 6'-Acetyl liquiritin

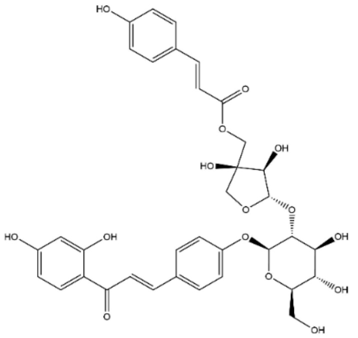
30. Licorice glycoside B

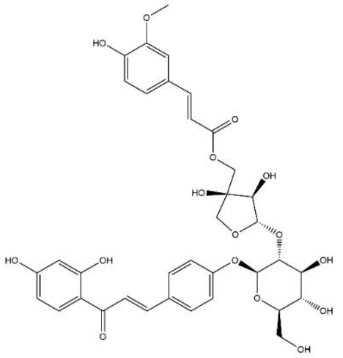
31. Licorice glycoside A

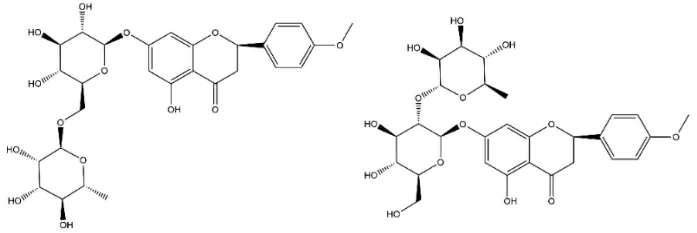
32. Didymin or Poncirin

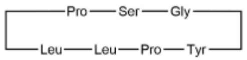

33. Citrusin III

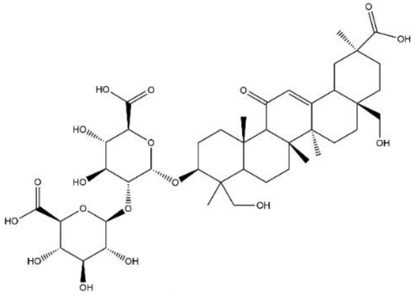
34. Dihydroxy glycyrrhizic acid

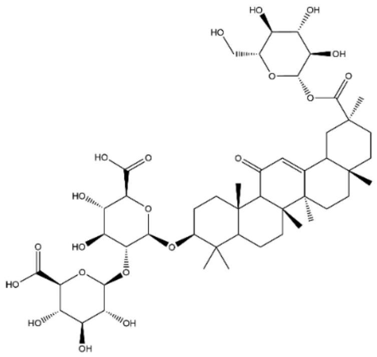
35. Licorice saponin A3

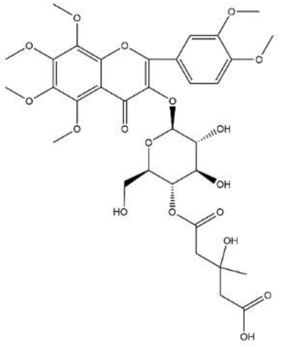
36. Natsudaidain 3-(4-O-3-hydroxy-3-methylglutaroylglucoside)

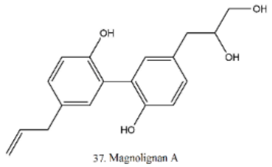
37. Magnolignan A

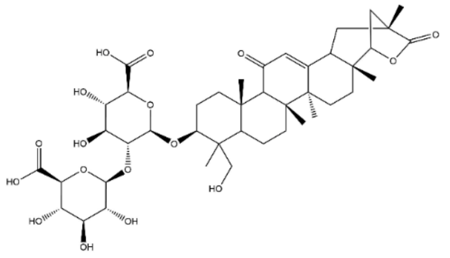

38. 24-Hydroxy-3beta-[(2-O-beta-D-glucopyranuronosyl-beta-D-glucopyranuronosyl) oxy]-11-oxo-22beta-hydroxyoleana-12-ene-30-oic acid gamma-lactone

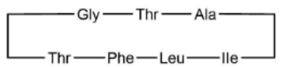

39. Citrusin I

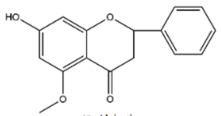

40. Alpinetin

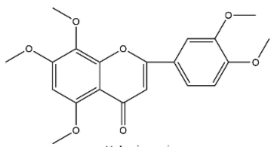

41. Isosinensetin

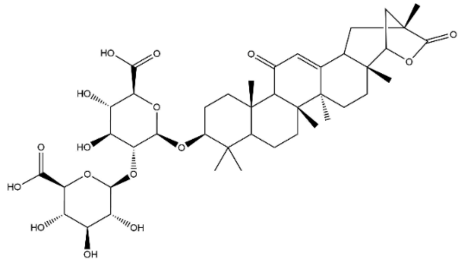

42. Licorice saponin E2

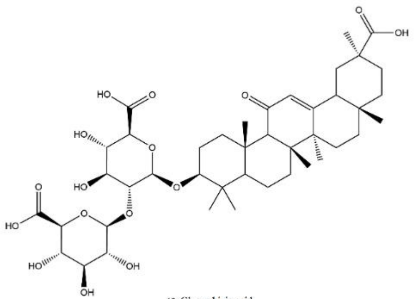

43. Glycyrrhizic acid

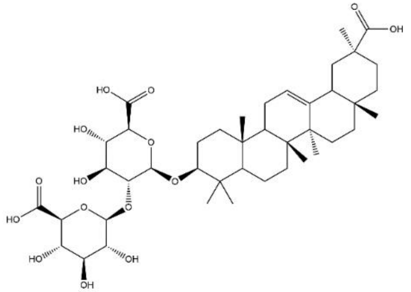

44. Licorice saponin B2

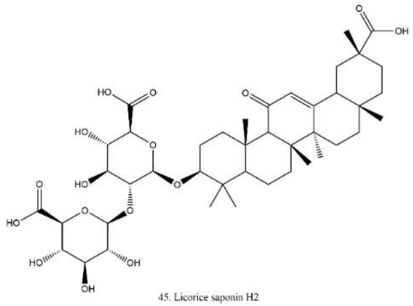

45. Licorice saponin H2

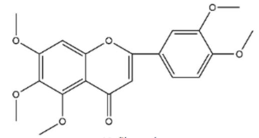

46. Sinensetin

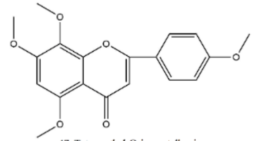

47. Tetramethyl-O-isoscutellarein

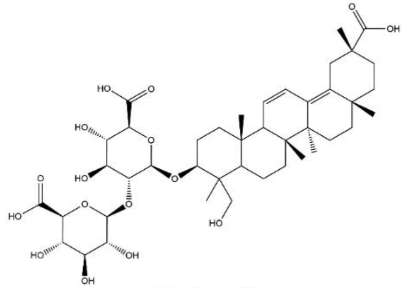

48. Licorice saponin K2

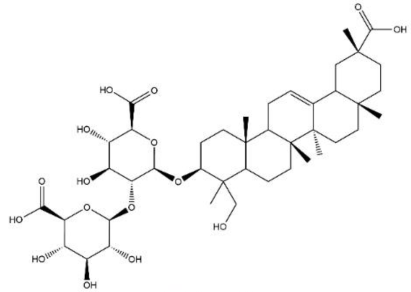

49. Licorice saponin J2

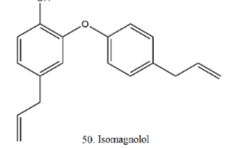

50. Isomagnolol

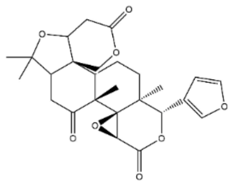

51. Limonin

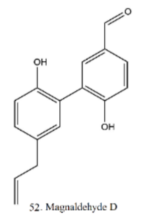

52. Magnaldehyde D

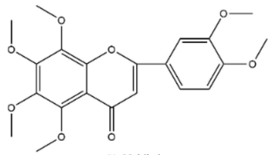

53. Nobiletin

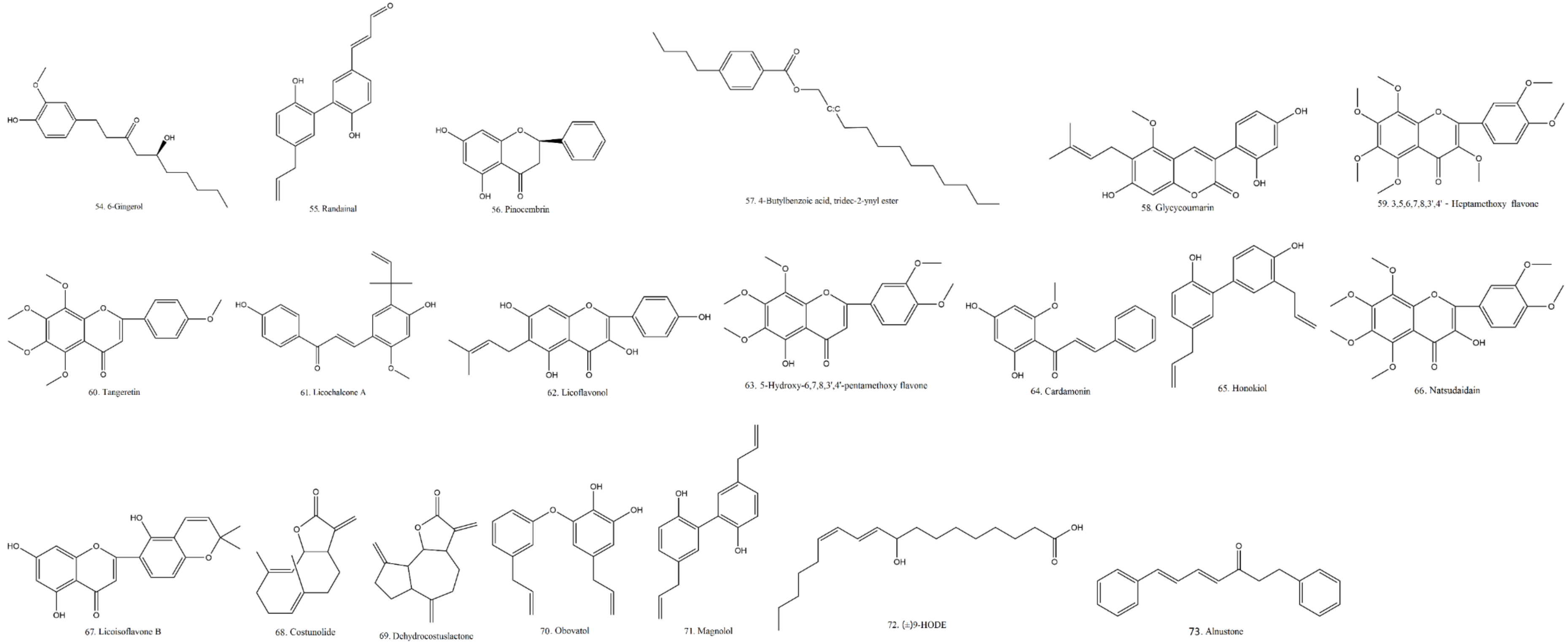

Figure S1. Structural Formulas of 73 Chemical Constituents.

Table S2. Detailed information on 88 chemical constituents of HWD and seven drugs.

Peak	RT (min)	Category	Compound	Mocular Formula	Molecular Mass	[M+H]+ (m/z)	[M+H]+ (m/z) (Δ ppm)	MSn Fragment Ions	[M-H]- (m/z)	[M–H]– (m/z) (Δ ppm)	MSn Fragment Ions	Refs
1	27.43	Alkaloids	Magnocurarine or Lotusine	$C_{19}H_{24}NO_3^+$	314.17507	314.17426	-2.578	271.13245, 270.12021, 269.11679, 237.09068, 175.07504, 149.02299				[32]
2	35.11	Flavonoids	Luteolin-6,8-di-C-glucoside	$C_{27}H_{30}O_{16}$	610.15284	611.15973	-1.523	593.18604, 575.35278, 557.18744, 473.26453, 353.22748	609.14612	1.820	489.11737, 399.15558, 369.09888	[33]
3	36.99	Alkaloids	Tembetarine	$C_{20}H_{26}NO_4^+$	344.18563	344.18550	-0.392	299.10132, 175.02397, 235.02451, 151.01923, 137.05937				[32]
4	38.80	Alkaloids	Magnoflorine	$C_{20}H_{24}NO_4^+$	342.16998	342.16946	-1.533	297.11176, 282.08862, 265.08575, 237.09088				[32]
5	41.37	Flavonoids	Vicenin-2	$C_{27}H_{30}O_{15}$	594.15792	595.16473	-1.708	617.14673, 596.16821, 618.14996, 577.15454, 437.23495, 301.14063, 149.02299	593.15106	1.624	248.96034, 242.94347, 215.00986	[34]

6	44.11	Alkaloids	Reticuline	$C_{19}H_{23}NO_4$	329.16216	330.16940	-1.771	301.14059, 299.12735, 271.26276, 247.05972, 192.10161, 163.03871, 150.02634, 149.02296, 137.05939, 136.02122				[32]
7	45.71	Flavonoids	Diosmetin 6,8-di-C-glucoside	$C_{28}H_{32}O_{16}$	624.16849	625.17493	-2.209	626.17847, 647.15686, 648.16040, 445.11899, 355.06915, 301.14035	623.16187	1.940		[35]
8	45.77	Phenylpropanoids	Magnoloside B	$C_{35}H_{46}O_{20}$	786.25770				785.25043	0.713	215.00966, 649.34503, 623.30731, 477.22992	[32]
9	48.45	Flavonoids	Chrysoeriol 6,8-di-C-glucoside	$C_{28}H_{32}O_{16}$	624.16849	625.17542	-1.426	647.15747, 648.16089, 626.17889, 445.11951, 355.06955, 301.14063				[35]
10	50.82	Flavonoids	Schaftoside	$C_{26}H_{28}O_{14}$	564.14736	565.15497	-0.375	587.13690, 403.13608, 271.26309	563.14081	2.270	545.20935, 503.27216, 473.18585, 443.71747, 425.22198, 413.17181, 383.12177, 353.13043	[36], [37], [37]

11	52.92	Phenylpropanoids	Magnoloside A	$C_{29}H_{36}O_{15}$	624.20487				623.19769	1.03226	461.22223, 315.28967	[38]
12	54.92	Alkaloids	Asimilobine	$C_{17}H_{17}O_2N$	267.12538	268.13275	-1.698	251.10626, 236.08293, 219.08015, 191.08522				
13	55.67	Flavonoids	Glucoliquiritin apioside	$C_{32}H_{40}O_{18}$	712.22092	713.22858	-0.225	735.21588, 569.31350, 257.08054	711.21375	0.927	417.11877, 255.06598	[37], [39]
14	56.36	Flavonoids	Liquiritin	$C_{21}H_{22}O_9$	418.12583	419.13321	-1.071	441.11447, 257.08035, 163.0381, 147.04376	417.11902	2.425	417.11902, 255.06601, 153.01935, 135.00897, 119.05056	[40], [41]
15	57.72	Flavonoids	Rutin	$C_{27}H_{30}O_{16}$	610.15284	611.16003	-1.033	593.33093, 575.13361, 557.18365, 473.12170, 353.34692	609.14563	1.016		[33]
16	58.41	Flavonoids	Luteolin-7-rutinoside	$C_{27}H_{30}O_{15}$	594.15792	595.16486	-1.490	449.12097, 287.12695	593.15051	0.697	443.17529, 285.02246	[34]
17	59.27	Flavonoids	Eriocitrin	$C_{27}H_{32}O_{15}$	596.17357				595.16656	1.367		[35]
18	59.88	Alkaloids	Xanthoplanine	$C_{21}H_{26}O_4N^+$	356.18563	356.18469	-2.653	311.12698, 296.10706, 280.13892				[42]
19	61.84	Phenylpropanoids	Magnoloside M	$C_{29}H_{36}O_{16}$	625.20487				623.19745	1.03226	461.32123, 315.11142	[42]
20	62.19	Flavonoids	Naringin	$C_{27}H_{32}O_{14}$	580.17866	581.18512	-2.343	603.16656, 582.18866, 419.13254, 435.12738, 273.07501	579.17133	0.860	271.06073	[43]

21	64.67	Phenylpropanoids	Verbascoside	$C_{29}H_{36}O_{15}$	624.20487				623.19684	-0.332	215.00951, 461.27814, 315.15555	[42]
22	64.85	Flavonoids	Hesperidin	$C_{28}H_{34}O_{15}$	610.18922	611.19647	-0.944	633.17767, 449.14334, 303.08575, 465.13812, 431.13306, 413.12250	609.18201	1.007	301.07135	[44]
23	66.23	Flavonoids	Limocitrin-3-O-(3-hydroxy-3-methylglut-arate)-glucoside or isomers	$C_{29}H_{32}O_{17}$	652.16340	653.17053	-1.065	675.15210, 347.07553	651.15643	1.312	549.12494	[35]
24	67.71	Flavonoids	Liguiritigenin-7-O-D-apiosyl-4'-O-D-glucoside	$C_{26}H_{30}O_{13}$	550.16809	551.17462	-2.354	257.08026	549.16119	1.680	417.15961, 255.16119	[36]
25	68.63	Flavonoids	Liquiritin apioside	$C_{26}H_{30}O_{13}$	550.16809	551.17566	-0.467	419.13327, 257.08066	549.16113	1.571	255.04315	[40], [45], [46]
26	68.78	Flavonoids	Ononin	$C_{22}H_{22}O_{9}$	430.12583	431.13324	-0.971	453.11487, 269.08044				[37].
27	69.01	Flavonoids	Isoliquiritin	$C_{21}H_{22}O_{9}$	418.12583	419.13327	-0.927	257.03751	417.11896	2.281	255.04541	[40], [45], [47]
28	69.55	Phenylpropanoids	Magnolignan C	$C_{18}H_{20}O_{4}$	300.13561				299.12866	2.923	239.00793, 221.12096, 174.95628, 146.96623, 144.00957	[48]
29	69.93	Flavonoids	6'-Acetyl liquiritin	$C_{23}H_{24}O_{10}$	460.13640				459.12949	1.997	417.15088, 399.29071, 255.03796, 297.11273, 137.01698	[37]

30	70.16	Flavonoids	Licorice glycoside B	$C_{35}H_{36}O_{15}$	696.20487	697.21228	-0.598		695.19714	0.134	549.16119, 531.15070, 417.11884, 255.06584	[37], [49]
31	71.33	Flavonoids	Licorice glycoside A	$C_{36}H_{38}O_{16}$	726.21544				725.20758	-0.043	695.19733, 549.27191, 531.20392, 399.23151, 255.12831	[37], [47]
32	72.48	Flavonoids	Didymin or Poncirin	$C_{28}H_{34}O_{14}$	594.19431	595.20166	-0.793	617.18329, 449.14359, 433.14874				[35]
33	74.25	Alkaloids	Citrusin III	$C_{36}H_{53}N_7O_9$	727.38993	728.39697	-1.074	700.54614, 587.42694,474.33710	726.38232	0.299		[50]
34	74.42	Triterpenoid saponins	Dihydroxy glycyrrhizic acid	$C_{42}H_{62}O_{18}$	854.39307	855.40045	-0.516	679.29767, 503.38794, 467.33405	853.38538	0.163		[36]
35	74.61	Triterpenoid saponins	Licorice saponin A3	$C_{48}H_{72}O_{21}$	984.45606	985.46381	-0.077	1007.44531, 965.29164, 647.37854, 453.33566	983.44910	0.879		[40], [49]
36	75.66	Flavonoids	Natsudaidain 3-(4-O-3-hydroxy-3-methylglutaroylglucoside)	$C_{33}H_{40}O_{18}$	724.22092	725.22772	-1.407	747.20917, 726.23114, 419.13263	723.21362	0.73200	417.11871	[35]
37	76.57	Phenylpropanoids	Magnolignan A	$C_{18}H_{20}O_4$	300.13561				299.12866	2.92300	239.06659, 221.09616, 146.96605, 144.00941	[48]
38	77.10	Triterpenoid saponins	24-Hydroxy-3beta-[(2-O-beta-D-glucopyranuronosyl-beta-D-	$C_{42}H_{60}O_{17}$	836.38250	837.39026	-0.080		835.37482	0.17200	659.44513, 351.08698	[45]

			glucopyranuronosyl) oxy]-11-oxo-22beta-hydroxyoleana-12-ene-30-oic acid gamma-lactone									
39	78.06	Alkaloids	Citrusin I	$C_{34}H_{53}N_7O_9$	703.38993	704.39673	-1.452	726.37848, 686.47504,555.23513				[33]
40	78.47	Flavonoids	Alpinetin	$C_{16}H_{14}O_4$	270.08866	271.09583	-2.418	293.07755, 167.03352, 131.04883, 103.05396, 90.88628	269.08139	2.06100		
41	79.67	Flavonoids	Isosinensetin	$C_{20}H_{20}O_7$	372.12035	373.12753	-1.740	395.10941, 343.15536, 358.14450, 329.06317				[33]
42	79.73	Triterpenoid saponins	Licorice saponin E2	$C_{42}H_{60}O_{16}$	820.38759				819.38019	0.52200	643.54840, 351.16757	[39],
43	80.62	Triterpenoid saponins	Glycyrrhizic acid	$C_{42}H_{62}O_{16}$	822.40324	823.41040	-0.804	845.39178, 647.37793, 471.34567, 453.33508	821.39618	0.93500	351.09686, 645.56256, 803.50635	[40],
44	82.08	Triterpenoid saponins	Licorice saponin B2	$C_{42}H_{64}O_{15}$	808.42397	809.43073	-1.319	831.41229, 633.39856, 457.36639	807.41681	0.821	351.12073, 631.41876	[36], [46]
45	82.47	Triterpenoid saponins	Licorice saponin H2	$C_{42}H_{62}O_{16}$	822.40324	823.41034	-0.877	845.39154, 647.37775, 471.34552, 453.33484				[40], [36], [45], [37].
46	82.61	Flavonoids	Sinensetin	$C_{20}H_{20}O_7$	372.12035	373.12766	-1.392	395.10947, 358.20599, 343.22675, 312.09213,				[33]

								329.10526, 340.15942				
47	82.78	Flavonoids	Tetramethyl-O-isoscutellarein	$C_{19}H_{18}O_6$	342.10979	343.11725	-1.063	365.09909, 344.12064, 313.07040, 250.17741				[51]
48	83.20	Triterpenoid saponins	Licorice saponin K2	$C_{42}H_{62}O_{16}$	822.40324	823.41040	-0.804	845.39203, 647.37817, 471.34604, 453.33545	821.39581	0.485	803.57916, 645.48639, 469.36035, 351.07977	[40], [36], [45], [37].
49	83.81	Triterpenoid saponins	Licorice saponin J2	$C_{42}H_{64}O_{16}$	824.41889	825.42584	-1.057	847.40717, 453.33521				[40], [52]
50	83.82	Phenylpropanoids	Isomagnolol	$C_{18}H_{18}O_2$	266.13013				265.12311	3.031	216.01256, 215.00948, 174.95593, 146.96590, 144.00929	[53]
51	84.09	Lactones	Limonin	$C_{26}H_{30}O_8$	470.19352	471.20056	-1.664	453.23090, 425.28485, 367.16846, 161.02158	469.18668	2.101		[33]
52	84.96	Phenylpropanoids	Magnaldehyde D	$C_{16}H_{14}O_3$	254.09375				253.08691	3.909	235.07635, 215.00981, 174.95621	[48]
53	85.60	Flavonoids	Nobiletin	$C_{21}H_{22}O_8$	402.13092	403.13824	-1.250	425.11978, 827.25214, 373.09167, 388.11517				[43]
54	86.05	Aromatic hydrocarbon	4-Butylbenzoic acid, tridec-2-ynyl ester	$C_{24}H_{36}O_2$	356.27098	357.27814	-1.867	339.28171, 321.26703, 275.16193				
55	86.20	Aromatic hydrocarbon	6-Gingerol	$C_{17}H_{26}O_4$	294.18256	295.17346	-1.736	317.17178, 277.17944,				[54], [55]

								259.16885, 177.09056				
56	86.94	Phenylpropanoids	Randainal	$C_{18}H_{16}O_3$	280.10940				279.10257	3.580	261.09186, 215.00964, 248.96021, 242.94332, 261.08942, 233.17661	[38]
57	86.99	Flavonoids	Pinocembrin	$C_{15}H_{12}O_4$	256.07301	257.08026	-2.238	152.87318, 130.89072, 102.90588, 90.97833	255.06569	1.979		[56]
58	87.30	Phenylpropanoids	Glycycoumarin	$C_{21}H_{20}O_6$	368.12544	369.13278	-1.313	341.23120, 327.17776, 313.17673, 285.09848				[40], [47]
59	87.60	Flavonoids	3,5,6,7,8,3',4'－Heptamethoxy flavone	$C_{22}H_{24}O_9$	432.14148	433.14856	-1.729	455.12944, 887.27319, 888.27643, 418.12561, 403.10217				[51]
60	89.06	Flavonoids	Tangeretin	$C_{20}H_{20}O_7$	372.12035	373.12766	-1.392	395.10941, 767.23053, 358.15698, 343.12009, 325.12558				[44]
61	90.46	Flavonoids	Licochalcone A	$C_{21}H_{22}O_4$	338.15126	339.15884	-0.724	297.12061, 271.06317, 245.11261	337.14404	1.793	187.08160	[49]
62	90.75	Flavonoids	Licoflavonol	$C_{20}H_{18}O_6$	354.10979				353.10281	2.394	285.18896	[45], [46]
63	92.06	Flavonoids	5-Hydroxy-6,7,8,3',4'-pentamethoxy flavone	$C_{20}H_{20}O_8$	388.11527	389.12262	-1.218	411.09378, 518.32318, 352.33905,				[33]

								335.21893, 163.03874, 149.02303				
64	92.31	Flavonoids	Cardamonin	$C_{16}H_{14}O_4$	270.08866	271.09607	-1.532	167.03363, 131.04889, 103.01764, 90.97807	269.08170	3.213	254.09070, 226.02644, 165.07042, 138.90138	
65	93.86	Phenylpropa noids	Honokiol	$C_{18}H_{18}O_2$	266.13013				265.12305	2.805	250.09950, 224.08372, 215.00948	[38]
66	93.96	Flavonoids	Natsudaidain	$C_{21}H_{22}O_9$	418.12583	419.13315	-1.214	411.09329, 371.10098, 359.24005, 352.33914, 149.02307				[35]
67	95.05	Flavonoids	Licoisoflavone B	$C_{20}H_{16}O_6$	352.09414	353.10150	-1.316	233.15326, 187.14774	351.08694	1.781	283.10434, 265.07629	[46]
68	95.13	Lactones	Costunolide	$C_{15}H_{20}O_2$	232.14578	233.15343	-0.757	215.14285, 187.14786, 145.10101, 131.08530				[57], [41]
69	96.37	Lactones	Dehydrocostuslacton e	$C_{15}H_{18}O_2$	230.13013	231.13770	-1.109	213.12708, 185.13211, 157.10095, 143.08527				[57], [41]
70	96.67	Phenylpropa noids	Obovatol	$C_{18}H_{18}O_3$	282.12505				281.11816	3.341	240.04395, 164.02255, 133.04956	[42]
71	97.24	Phenylpropa noids	Magnolol	$C_{18}H_{18}O_2$	266.13013				265.12259	1.070	247.11214, 245.09665, 215.00925	[38]
72	99.48	Fatty acids	(±) 9-HODE	$C_{18}H_{32}O_3$	296.23460				295.22760	2.807	265.12311, 277.21689, 195.13872	[48]

73	102.41	Ketones	Alnustone	$C_{19}H_{18}O$	262.13522	263.14249	-2.097	171.98802, 157.01140, 147.05292, 133.06444, 129.01965, 116.90790, 105.06956, 91.00671
